# The need to return to the basics of predictive modelling for disease outbreak response: lessons from COVID-19

**DOI:** 10.11604/pamj.2020.36.355.24101

**Published:** 2020-08-27

**Authors:** Sam Agatre Okuonzi

**Affiliations:** 1 Arua Regional Referral Hospital, Arua, Uganda

**Keywords:** Modelling, context, Africa

## Abstract

The outbreak of COVID-19 has been unprecedented in speed and effect. Efforts to predict the disease transmission have mostly been done using flagship models developed by the global north. These models have not accurately depicted the true rate of transmission of SARS-CoV-2 in Africa. The models have ignored Africa’s unique socio-ecological makeup (demographic, social, environmental and biological) that has aided a slower and less severe spread of the virus. This paper opines on how the science of infectious disease modelling needs to evolve to accommodate contextual factors. Country-owned and tailored modelling needs to be urgently supported.

## Commentary

The outbreak of Coronavirus Disease of 2019 (COVID-19) pandemic has been unprecedented. Caused by Severe Acute Respiratory Syndrome Coronavirus Type 2 (SARS-CoV-2) it started in China in 2019 and spread to almost all countries of the world within three months. This has underscored the need for strong monitoring of preparedness and response actions across countries. The pandemic has, almost simultaneously, affected all countries of the world but with significantly different outcomes. These outcomes bear no relationship to the current assessed health security capacities. The numbers of cases and deaths are not related to country International Health Regulations (IHR) core capacity scores [1, 2], nor are they related to the performance of global health security index [3]. This is contrary to the expectation that countries with good IHR and global health security scores would be more successful at controlling the outbreak and mitigating its effects. In most instances, evidence of the converse has been observed [4]. Why is this so?

**Doomsday prophecy:** at the beginning of the outbreak, there was a near universal concern for the countries perceived to be poor, with low IHR scores and with weak health systems. Initial disease predictive models were doomsday prophesies as they painted a picture of devastation of lives and economies, particularly in the African Region [5, 6]. The rate of transmission and severity of the pandemic have however not played out in line with these predictions, despite the fact that the pandemic is now five months old. A more recent predictive model from the WHO Regional Office for Africa seems to explain why this is so. This was done by appropriately contextualizing the outbreak into the unique socio-ecological make up of countries [7]. Analyses that do not consider these seem to predict higher and more severe events, which have so far not been the case in any country of the Africa Region [8-10]. Infectious disease modelling has traditionally focused on pathogen transmission characteristics, often making the simplistic assumption that the application of a minor correction factor could account for the social, environmental, and biological interactions that influence transmission. However, in our increasingly globalized world, pathogens rapidly move across varying socio-ecological environments, expressing differently, and spreading at different speeds and with different severity in these different environments. Predictive models that do not try to understand and factor in this critical dynamic often end up being inaccurate, with gross over-estimations.

**The reality:** this socio-ecological environment that has been observed to influence the SARS-CoV-2 spread within and across countries is rather complex. It encompasses social, demographic, cultural, environmental, and biological factors that interact with each other in unique ways to influence rates of transmission and effects on individuals. The lessons from this calls for subsequent modelling to consider two key attributes: (1) what factors to include, and (2) how they interact, in order to understand their influence on transmission and outcome patterns. For instance, assuming high population density alone affects rate of transmission, this does not explain the relatively lower rate of SARS-CoV-2 transmission in India [4]. Assuming only high precipitation rates reduce the rate of transmission, this ignores the high rates of transmission observed in Brazil and Indonesia. Focusing solely on age ignores the low rates of severe disease in Japan-a country with the oldest population. Considering only environmental factors as drivers of transmission would ignore the differential transmission observed in countries such as Iraq/Iran, Dominican Republic/Haiti, Indonesia/Malaysia, USA/Canada and South Africa/Botswana. In the development of a good predictive model, one needs to take into account all these factors that influence disease spread and severity, and incorporate how they interact.

**Pseudo-science:** this lack of contextualization is turning predictive modelling into a pseudo-science. It has turned to producing large, unrealistic estimates of disease effects, often targeting donor audiences. This makes it difficult for governments to take rational actions for planning and resource allocation. This kind of donor-driven estimates of disease leads to wasteful investments, such as tent hospitals. This is tragic as resources in Africa are particularly scarce. The opportunity cost of investing in initiatives that will not help the population is devastating. From example, African governments have been purchasing ventilators, though they lack the resources to use them. They have been designating COVID-19 hospitals and treatment centres despite the fact that most patients have only mild symptoms. These patients can be managed within the existing service delivery framework.

**Overzealous donors:** the observed behaviour of donors has been to make more money available, with the International Monetary Fund (IMF) and World Bank providing countries with response loans [11, 12]. The Global Fund and others have increased country level grant flexibilities to accommodate expenditure on COVID response efforts [13]. These external efforts were implemented without a recognition of the fact that most of the interventions would be domestically funded. The risk of misappropriation of external funding and poor management of resources for service provision would have been abated if more context-specific predictive modelling techniques had been used to guide policy actions. The inability to contextualize predictions is harming the overall science of predictive modelling. It is shifting from the detective art that epidemiology is, to a commercialized mechanical process. Teams with the most powerful technologies are the ones that get heard instead of those with models that reflect country-specific characteristics. At present, a few centres-the ones with the best computing power-have become self-appointed predictive modelling gate-keepers and make it difficult for new entrants.

## Conclusion

Given the scepticism that has surrounded the “flagship” models of this pandemic, as a result of their over-estimation, a number of countries in Africa have turned to their own academic institutions for guidance on modelling results. This is a good first step. However, these academic institutions still rely on the gate-keepers for methodologies, instead of developing novel ways of building predictive models that would respond to their specific needs. As countries accelerate efforts to decolonize global health, it would be crucial to accelerate local approaches to disease modelling. In particular, to develop a better understanding and application of socio-ecological and environmental drivers of disease transmission and outcomes. WHO-AFRO model provides a good basis to reinstate this important epidemiological tool.
